# Mesenchymal stem cell–derived conditioned medium attenuate angiotensin II‐induced aortic aneurysm growth by modulating macrophage polarization

**DOI:** 10.1111/jcmm.14694

**Published:** 2019-10-04

**Authors:** Yang‐zhao Zhou, Zhao Cheng, Yin Wu, Qi‐ying Wu, Xiao‐bo Liao, Yuan Zhao, Jian‐ming Li, Xin‐min Zhou, Xian‐ming Fu

**Affiliations:** ^1^ Department of Cardiovascular Surgery The Second Xiang‐ya Hospital Central South University Changsha China; ^2^ Department of Hematology The Second Xiang‐ya Hospital Central South University Changsha China

**Keywords:** aortic aneurysm, conditioned medium, macrophage polarization, matrix metalloproteinases, mesenchymal stem cells

## Abstract

Mesenchymal stem cells (MSCs) exhibit therapeutic benefits on aortic aneurysm (AA); however, the molecular mechanisms are not fully understood. The current study aimed to investigate the therapeutic effects and potential mechanisms of murine bone marrow MSC (BM‐MSCs)–derived conditioned medium (MSCs‐CM) on angiotensin II (AngII)‐induced AA in apolipoprotein E‐deficient (apoE^−/−^) mice. Murine BM‐MSCs, MSCs‐CM or control medium were intravenously administrated into AngII‐induced AA in apoE^−/−^ mice. Mice were sacrificed at 2 weeks after injection. BM‐MSCs and MSCs‐CM significantly attenuated matrix metalloproteinase (MMP)‐2 and MMP‐9 expression, aortic elastin degradation and AA growth at the site of AA. These treatments with BM‐MSCs and MSCs‐CM also decreased Ly6c^high^ monocytes in peripheral blood on day 7 and M1 macrophage infiltration in AA tissues on day 14, whereas they increased M2 macrophages. In addition, BM‐MSCs and MSCs‐CM reduced MCP‐1, IL‐1Ra and IL‐6 expression and increased IL‐10 expression in AA tissues. In vitro, peritoneal macrophages were co‐cultured with BM‐MSCs or fibroblasts as control in a transwell system. The mRNA and protein expression of M2 macrophage markers were evaluated. IL‐6 and IL‐1β were reduced, while IL‐10 was increased in the BM‐MSC systems. The mRNA and protein expression of M2 markers were up‐regulated in the BM‐MSC systems. Furthermore, high concentration of IGF1, VEGF and TGF‐β1 was detected in MSCs‐CM. Our results suggest that MSCs‐CM could prevent AA growth potentially through regulating macrophage polarization. These results may provide a new insight into the mechanisms of BM‐MSCs in the therapy of AA.

## 1 INTRODUCTION

Aortic aneurysm (AA) is a chronic aortic disease characterized by the permanent dilation of aortic wall with a risk of aortic rupture. Although significant advances have been made in open surgery and endovascular repair, no medical therapies are available to prevent AA growth.^1^, ^2^ The underlying mechanisms of AA formation and progression were involved in chronic inflammatory response, matrix metalloproteinases (MMPs) up‐regulation, extracellular matrix (ECM) degradation and apoptosis of vascular smooth muscle cells (VSMCs). Macrophages have been identified in AA tissues and play vital roles in expression of pro‐inflammatory cytokines and activation of MMPs that contribute to AA development.^3^

Macrophages are divided into two phenotypes including ‘classically activated’ M1 macrophages and ‘alternatively activated’ M2 macrophages. M1 macrophages exhibit pro‐inflammatory activities through producing pro‐inflammatory cytokines and proteolytic enzymes, whereas M2 macrophages have anti‐inflammatory/reparative effects through secretion of factors such as IL‐10 or TGF‐β and contribute to resolution of inflammation and tissue remodelling. iNOS and CD206 are commonly used to identify M1 and M2 macrophages, respectively.^4^ Recent findings demonstrated that macrophages play an important role in the modulation of vascular inflammation and aortic remodelling. The rapamycin inhibitor everolimus limited angiotensin II (AngII)‐induced AA formation in apolipoprotein E‐deficient (apoE^−/−^) mice via suppressed development of bone marrow CCR2 monocytes and diminished M1 polarization.^5^ D‐series resolvins could attenuate inflammatory activities and AA formation by increasing M2 macrophage polarization.^6^ Elastin‐derived peptides (EDPs) promoted a pro‐inflammatory environment in aortic tissues and attenuated aortic dilation through induction of M1 polarization, and neutralization of EDPs. Furthermore, injection of M2‐polarized macrophages reduced AA expansion.^7^ According to a recent study, CD31 agonist P8RI induced M2 macrophage polarization and promoted the healing of experimental dissected AA in apo E^−/−^ mice.^8^ This evidence indicates that macrophage polarization might be a potential target for the treatment of AA.

Bone marrow–derived mesenchymal stem cells (BM‐MSCs) exert immunosuppressive effects and represent a promising treatment in regenerative medicine. Increasing evidence supports that MSCs provide the therapeutic potential to inhibit AA progression via paracrine effect.^9^, ^10^, ^11^, ^12^ BM‐MSC‐derived conditioned medium (MSCs‐CM) is thought to be a potent secretory component of BM‐MSCs containing specific cytokines, growth factors and small RNAs, which play an important role in MSC‐mediated therapeutic effects.^13^ Several recent studies have demonstrated that administration of MSCs‐CM was as effective as BM‐MSCs treatment itself in animal models of tissue injury and inflammatory diseases.^14^, ^15^, ^16^, ^17^ Recent studies have disclosed that BM‐MSCs reduced inflammatory response and promoted tissue repair by inducing macrophage phenotype conversion from M1 to M2 in various disease models.^18^, ^19^, ^20^, ^21^, ^22^ Previously, Hashizume and colleagues have reported that BM‐MSCs attenuated AngII‐induced AA growth in apoE^−/−^ mice by surgically implanting BM‐MSCs sheet on the surface of aortic adventitial.^9^ Recently, our group have reported that intravenous administration of BM‐MSCs prevented AngII‐induced AA development in apoE^−/−^ mice.^23^, ^24^ These experiments demonstrated the effects of BM‐MSCs on the treatment of AA. Nonetheless, the underlying mechanisms are not fully understood and the effects of MSCs‐CM on the development of AA remain to be determined. Based on these previous studies, in the present study, we demonstrated that MSCs‐CM could effectively alleviate AngII‐induced AA growth in apoE^−/−^ mice compared with BM‐MSCs, and these protective effects contributed at least partially to modulating macrophage polarization.

## 2 MATERIALS AND METHODS

### 2.1 Animals

Eight‐week‐old male apoE^−/−^ mice were purchased from Beijing Huafukang Bioscience CO. INC. All apoE^−/−^ mice were cared for in accordance with the Guide of the Care and Use of Laboratory Animals published by the US National Institutes of Health (Publication No. 85‐23). All procedures and experiments were approved by the Animal Experiment Advisory Committee of the Second Xiang‐ya Hospital of Central South University.

### 2.2 Macrophages and BM‐MSC culturing

Bone marrow MSCs (BM‐MSCs) were isolated and cultured using standard protocols.^23^ The femurs from eight‐week‐old apoE^−/−^ mice were flushed with PBS to collect bone marrow cells. Cells were then cultured in T25 cell culture flasks in low glucose Dulbecco's modified Eagle's medium containing 15% FBS (Invitrogen) at 37°C with 5% CO_2_. After 4 days, culturing medium was changed to remove the non‐adherent cells and subsequently at three‐day intervals. The adherent cells were trypsinized with 0.25% trypsin‐EDTA (Invitrogen) and passaged into new flasks for further expansion when the cells reached 80%‐90% confluence; at passage 3‐5, BM‐MSCs were used for the detection of immunophenotype and multipotent differentiation potential. For murine embryonic fibroblast (MEF) isolation, uteri isolated from 13.5 days pregnant mice were collected and washed with PBS. The head and visceral tissue were removed from each embryo. The remainder of the bodies were washed in fresh PBS, minced with a pair of scissors, transferred into a 0.25‐mmol/L trypsin/ 1‐mmol/L EDTA solution (3 mL per embryo) and incubated at 37°C for 20 minutes. After incubation, an additional 3 mL PBS was added and then the mixture was incubated at 37°C for 20 minutes. After trypsinization, an equal amount of medium (6 mL per embryo of DMEM containing 10% FBS) was added and aspirated with a pipette to achieve tissue dissociation. Cells were filtered through a mesh, seeded at 1 × 10^6^ cells per 10‐cm tissue culturing dish and incubated at 37°C in 5% CO_2_. To prepare peritoneal macrophages, apoE^−/−^ mice were injected intraperitoneally with 1 mL of 3% thioglycollate medium (Sigma). After 3 days, cells were harvested from the peritoneal cavities by lavage.

### 2.3 Preparation of bone marrow MSCs‐CM

BM‐MSCs of passage 2‐3 were used to prepare the MSCs‐CM. Upon 70%‐80% of confluence, culture medium was removed and cells were washed with PBS before feeding with 12 mL of DMEM supplemented with 1% FBS and 1% penicillin/streptomycin. After 48 hours of incubation, the cell culture‐conditioned medium was collected and was centrifuged (8000 g, 5 minutes at 4°C) and filtered through a 0.2‐mm‐pore size filter to remove cell debris. Then, the conditioned medium was concentrated using ultrafiltration with a cut‐off of 10 kD (Millipore). The collected culture‐conditioned medium was defined as MSCs‐CM and was stored at −80°C before being used for the following experiments.

### 2.4 Establishment of AA model in apoE^−/−^ mice, control medium, BM‐MSCs or MSCs‐CM administration

**Figure 1:**
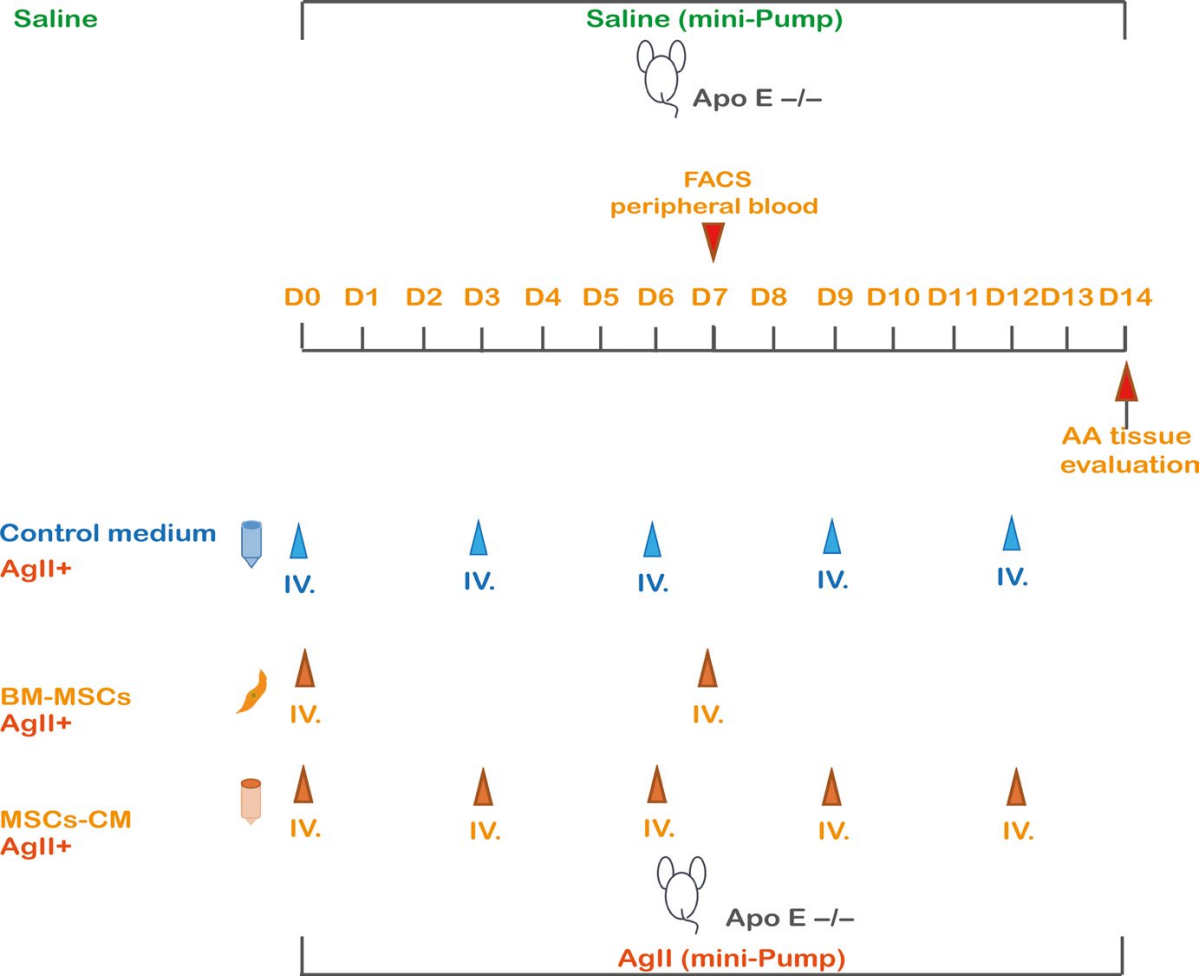
Schema of in vivo protocol. AA was induced by Ang II infusion for 2 wk in ApoE^−/−^ mice; 1 × 10^6^ BM‐MSCs (in 0.2 mL saline) were transplanted via tail vein on day 0, and day 7. 0.2 mL MSCs‐CM or 0.2 mL DMEM alone was injected via the tail vein on day 0, day 3, day 6, day 9 and day 12. Peripheral blood was collected on day 7 for flow cytometry. Instead of AngII administration, saline was used as sham. All mice were sacrificed, and AA tissue was evaluated on day 14

The ApoE^−/−^ mice were anaesthetized (0.1 mL/20 g, 10% chloral hydrate, i.p.). All mice were randomly divided into four groups (n = 8 per group): the saline group (mice with only saline infusion not AngII), control medium (DMEM supplemented with 1% FBS and 1% penicillin/streptomycin) group (mice with AngII infusion and control medium injection), BM‐MSCs group (mice with AngII infusion and BM‐MSCs injection) and MSCs‐CM group (mice with AngII infusion and MSCs‐CM injection; Figure 1). For AA induction, mice were infused with 1.44 mg/kg/d angiotensin II (Sigma‐Aldrich) that was subcutaneously delivered by an Alzet model 2004 osmotic minipump (DURECT Corp.). After infusion, 0.2 mL DMEM alone, 1 × 10^6^ BM‐MSCs (in 0.2 mL saline) or 0.2 mL concentrated MSCs‐CM was administrated via the tail vein. Mice were anaesthetized by isoflurane overdose. The length of the aorta from thoracic to abdominal cavity was exposed and photographed carefully using a LEICA DFC450C attached to a LEICA M165FC with the Leica Application Suite software (LAS 4.5.0). Digital image analysis software (ImageJ v.1.41, National Institutes of Health) was used to measure the maximum aortic diameter in the infra‐diaphragm by calibrating with ruler correction. Four‐millimetre lengths of the aorta with maximum diameter at the infra‐diaphragm was harvested for further investigations.

### 2.5 Measurement of elastin content in aortic tissue

The elastin amounts of the part of aorta with maximum diameter harvested from apoE^−/−^ mice were quantified using a Fastin elastin assay kit (Biocolor, County Antrim) as previously described.^23^

### 2.6 Flow cytometry

250‐300 μL of blood samples was collected for flow cytometry analysis on day 7 after AngII infusion. The following antibodies were used for staining: FITC antimouse Ly‐6C (Biolegend 128005), PerCP antimouse/human CD11b (Biolegend 101229) and PE antimouse CD45 (Biolegend 103105). Forward scatter (FSC) and side scatter (SSC) were used to gate live cells excluding red blood cells, debris and cell aggregates among total blood cells. Cells were acquired using a BD FACS Canto II (BD Biosciences) and analysed with FlowJo (Tree Star, Inc). The percentage of CD45^+^CD11b^+^ly6C^hi^ cells among the total leucocyte population were calculated and analysed.

### 2.7 Immunostaining

The part of aorta with maximum diameter were harvested on day 14 and embedded in Tissue‐Tek OCT compound (SAKURA 4583, Sakura Finetek, USA, Inc) The samples were frozen at −80°C. Then, 5‐um sections were fixed in pre‐cold 4% formaldehyde for 15‐20 minutes. The samples were blocked in 5% BSA in PBS for 1 hour. Next, Alexa 488 antimouse F4/80 (Biolegend 123119), antimouse NOS2 PE‐eFluor 610 (eBioscience) and Alexa Fluor 647 antimouse CD206 (MMR) (Biolegend 141711) antibodies were used to detect the expression of the M1 or M2 macrophage markers in the aortas. The incubations were performed at room temperature for 1.5 hour in a humidified box and protected from light. DAPI was used to stain the cell nuclei (blue) at a concentration of 1 µM. ProLong™ Gold Antifade Mountant (ThermoFisher, P36930) was applied to protect the fluorescence from fading. Images were taken using a Carl Zeiss Axio Imager AX10 with ZEN 2011 (blue edition). The fluorescence was measured using digital image analysis software (ImageJ v.1.41, National Institutes of Health). The percentage of iNOS^+^ and CD206^+^ cells relative to the F4/80^+^ cells was calculated and analysed in each group.

### 2.8 Aortic cytokine measurements

The part of aorta with maximum diameter were snap‐frozen and analysed using a Proteome Profiler Mouse Cytokine Array Panel A Kit (R&D Systems; catalogue number ARY006). The tissues were processed according to the manufacturer's protocol. The densitometric volume was determined by spectrophotometry using Thermo Scientific software (Thermo Fisher Scientific). All the procedures were performed strictly according to the manufacturers' instructions.

### 2.9 Direct or transwell co‐culture

For direct co‐culture experiments, 2 × 10^5^ macrophages were washed with PBS and 2 × 10^5^ BM‐MSCs or 2 × 10^5^ MEFs were plated directly in six‐well plate and cultured for 48 hours. For transwell co‐culturing, 1 × 10^5^ peritoneal macrophages were seeded into a six‐well plate. The next day, the 0.4‐mm‐pore size Corning transwell inserts (Sigma‐Aldrich) containing 2 × 10^5^ BM‐MSCs or 2 × 10^5^ MEFs were placed into the six‐well plate with the macrophages that were initially seeded.

### 2.10 Enzyme‐linked immunosorbent assay (measurement of supernatant cytokines)

The levels of insulin‐like growth factor 1 (IGF‐1) (MG100, USA R&D Systems, Inc), vascular endothelial growth factor (VEGF) (MMV00, USA R&D Systems, Inc), transforming growth factor beta 1 (TGF‐β1), (MB100B, USA R&D Systems, Inc), basic fibroblast growth factor (FGF basic) (MFB00, USA R&D Systems, Inc), stromal cell–derived factor 1 (SDF‐1α), (MCX120, USA R&D Systems, Inc) in the conditioned medium of BM‐MSCs and fibroblasts were determined by ELISA (n = 3). The secreted IL‐6 and IL‐1β in the supernatants of M1 phenotype, macrophage cells, direct co‐culturing M1 phenotype macrophage cells with BM‐MSCs/MEFs or transwell co‐cultured M1 phenotype macrophage cells with BM‐MSCs/MEFs were determined using IL‐6 Mouse ELISA Kit (Invitrogen, BMS603‐2, ThermoFisher scientific) and IL‐1β Mouse Uncoated ELISA Kit with Plates (Invitrogen, 88‐8014‐22, ThermoFisher scientific). The secreted IL‐10 in the supernatants of M2 phenotype macrophages, direct co‐culturing M1 M2 phenotype cells with BM‐MSCs/MEF or transwell co‐cultured M1 M2 phenotype cells with BM‐MSCs/MEF were determined using IL‐10 Mouse ELISA Kit (Invitrogen, BMS614‐2TWO, ThermoFisher scientific). All the procedures were performed strictly according to the instructions of manufactures.

### 2.11 RNA extraction, cDNA synthesis and qRT‐PCR

Total RNA was extracted from homogenized macrophage cells using the TaKaRa MiniBEST Universal RNA Extraction Kit (Takara, 9767) according to the manufacturer's instructions. cDNA was synthesized using PrimeScript™ RT reagent Kit with gDNA Eraser (Perfect Real Time) (Takara, RR047A). Real‐time PCR was performed using TB Green™ Premix Ex Taq™ II (Tli RNaseH Plus) (Takara RR820A) and LightCycler/LightCycler 480 System (Roche Diagnostics) according to the manufacturer's instructions. Expression levels of the target genes were normalized to that of beta‐actin. Primer sequences used in this study are listed in Table 1.

**Table 1:** The primers used for real‐time PCR

Genes	Forward (5′–3′)	Reverse (5′–3′)
CD206	AACGGAATGATTGTGTAGTTCTAGC	TACAGGATCAATAATTTTTGGCATT
Arg1	CCAGATGTACCAGGATTCTC	AGCAGGTAGCTGAAGGTCTC
Fizz1	GAATCTATTGTGGAGAAAAAGGTCA	AGCCGTGATACTAGTACAGGAGAAA
Ym1	GTGTACTCACCTGATCTATGCCTTT	CAGGAGAGTTTTTAGCTCAGTGTTC
TNFα	AACTCCAGGCGGTGCCTATG	TCCAGCTGCTCCTCCACTTG
IL‐6	AAGTCCGGAGAGGAGACTTC	TGGATGGTCTTGGTCCTTAG
IL‐1β	AGCTTCAGGCAGGCAGTATC	TCATCTCGGAGCCTGTAGTG
IL‐10	ACTCTTCACCTGCTCCACTG	GCTATGCTGCCTGCTCTTAC
MMP2	CCAGATGTGGCCAACTACAA	GGCATCATCCACTGTCTCTG
MMP9	GCAGAGATGCGTGGAGAGTC	ATGTTGTGGTGGTGCCACTT
β‐Actin	CACGAAACTACCTTCAACTCC	CATACTCCTGCTTGCTGATC

### 2.12 Western blotting

The Western blotting analysis was performed as previously described. Briefly, cells were harvested and homogenized on ice with RIPA (Beyotime Biotechnology P0013B) containing proteinase inhibitor (Complete, EDTA‐free protease inhibitor cocktail Tablets provided in EASY pack, Roche, 04 693 132 001). An equivalent amount of the cell lysate was subjected to sodium dodecyl sulphate‐polyacrylamide gel electrophoresis (SDS‐PAGE) and then transferred for the Western blotting assay. The polyvinylidene fluoride (PVDF) membranes were incubated with antimannose receptor antibody [EPR6828(B)] (CD206) (ab125028, Abcam), arginase‐1 (E4U1I) Mouse mAb (#43933, Cell Signaling Technology), β‐actin (8H10D10) mouse mAb (#3700, Cell Signaling Technology) primary antibodies and the appropriate horseradish peroxidase (HRP)–linked anti‐rabbit/mouse IgG secondary antibodies (Cell Signaling Technology) according to the primary antibody species. The membranes were treated with an enhanced chemiluminescent detection kit (Thermo Fisher Scientific, #17097), and the signals were collected and photographed (Bio‐Rad, Gel Doc, XR). All experiments were repeated three times. The bands were quantified by densitometry with the Scion Image software (ImageJ 1.42q; NIH).

### 2.13 Statistics analysis

The statistical significance of differences between groups was calculated by chi‐square test or unpaired *t* test, as appropriate, using GraphPad Prism 5.0 for Windows. The data are expressed as the mean ± SEM. Values were considered significantly different when *P* < .01.

## 3 RESULTS

### 3.1 MSCs‐CM attenuated AngII‐induced aortic aneurysm growth

**Figure 2:**
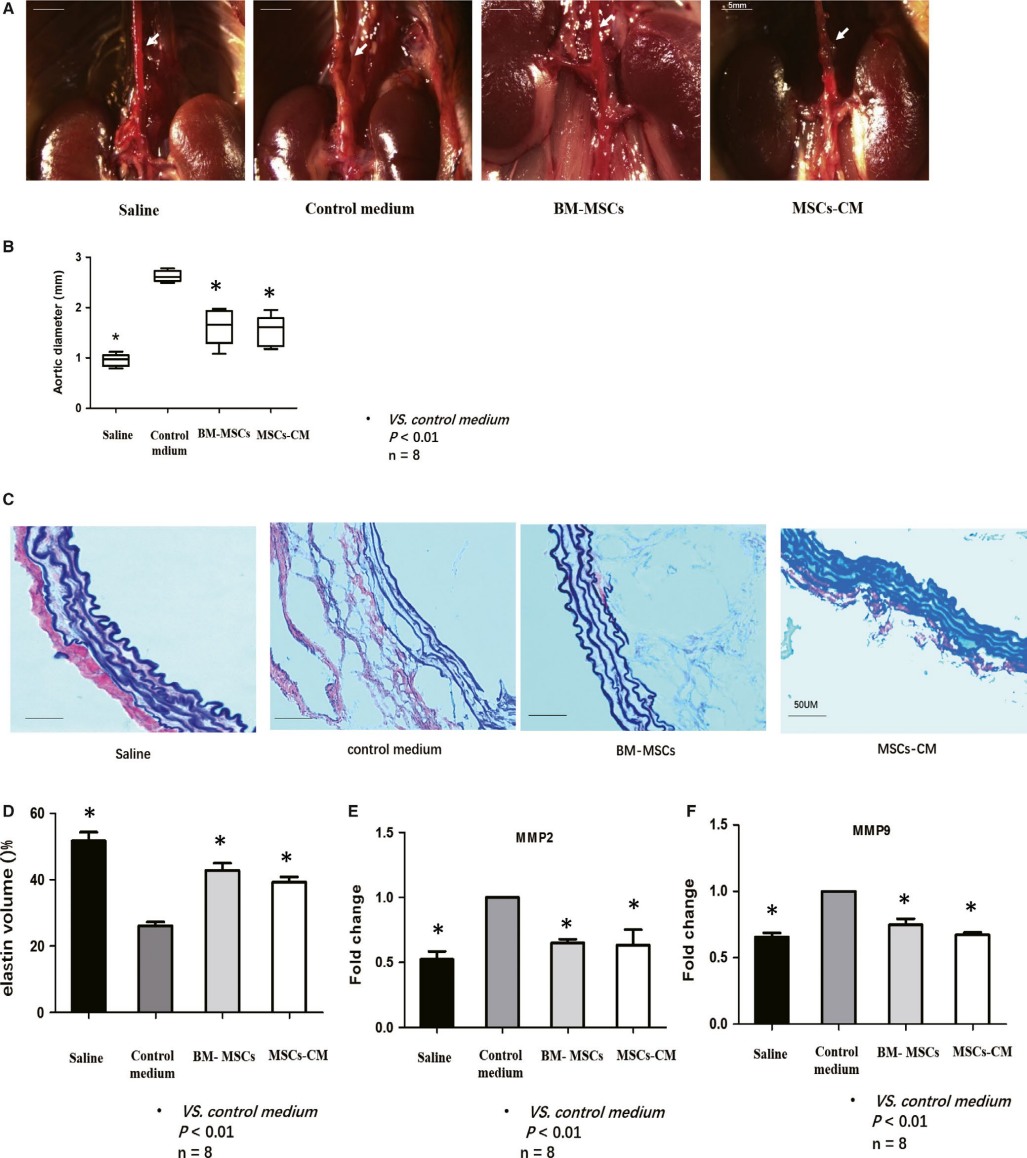
A, Representative images of AA in each group were presented. B, The maximum aortic diameter at the infra‐diaphragm was measured. BM‐MSCs and MSCs‐CM group showed reduced aortic diameter comparing with control medium group. C, Elastin structure of aortic wall was evaluated in each group using EVG staining. Elastin degradation was prevented in both BM‐MSCs and MSC‐CM group comparing with control medium group. D, Elastin volume of aortic wall was further evaluated in each group. Elastin volume of both BM‐MSCs and MSC‐CM group was higher than that of control medium group. E, F, mRNA expressions of MMP2 and MMP9 were determined using real‐time PCR. Fold changes were calculated and statistically analysed. MMP2 expression was significantly decreased in both BM‐MSC and MSCs‐CM group compared with control medium group; however, MMP9 expression was slightly decreased in both BM‐MSC and MSCs‐CM group

No deaths were observed in any of the mice in this study. The morphology of the aorta (maximum aortic diameter) in the saline, control medium, BM‐MSCs and MSCs‐CM groups are shown in Figure 2A. Typical AA could be observed in the control medium.

The mean of the maximum aortic diameter from the control medium group (2.626 ± 0.05 mm) was much larger than that from the BM‐MSCs group (1. 62 ± 0.06 mm) and the MSCs‐CM group (1.528 ± 0.13 mm; Figure 2B). These data indicated that both BM‐MSCs and MSCs‐CM could attenuate AngII‐induced AA progression.

### 3.2 BM‐MSCs and MSCs‐CM prevented AngII‐induced aortic elastin degradation and MMPs expression

Representative images of elastic lamellae using EVG staining from four groups are shown in Figure 2C. Considerable destruction of the elastic lamellae was observed in the control medium group, whereas less loss of the elastic lamellae was observed in both BM‐MSCs and MSCs‐CM group. Elastin volume was further determined in each group. Comparing with control medium group (26.11% ± 1.16), both BM‐MSCs (42.79% ± 2.18) and MSCs‐CM (34.96% ± 1.62) group showed preserved elastin volume (Figure 2D). MMP2 and MMP9 mRNA expressions were determined with real‐time PCR; significant decrease in MMP2 and MMP9 was observed in both BM‐MSCs and MSCs‐CM groups comparing with control medium group (Figure 2E,F).

### 3.3 BM‐MSCs and MSCs‐CM decreased subpopulation of CD45^+^CD11b^+^Ly6c^high^ monocytes

**Figure 3:**
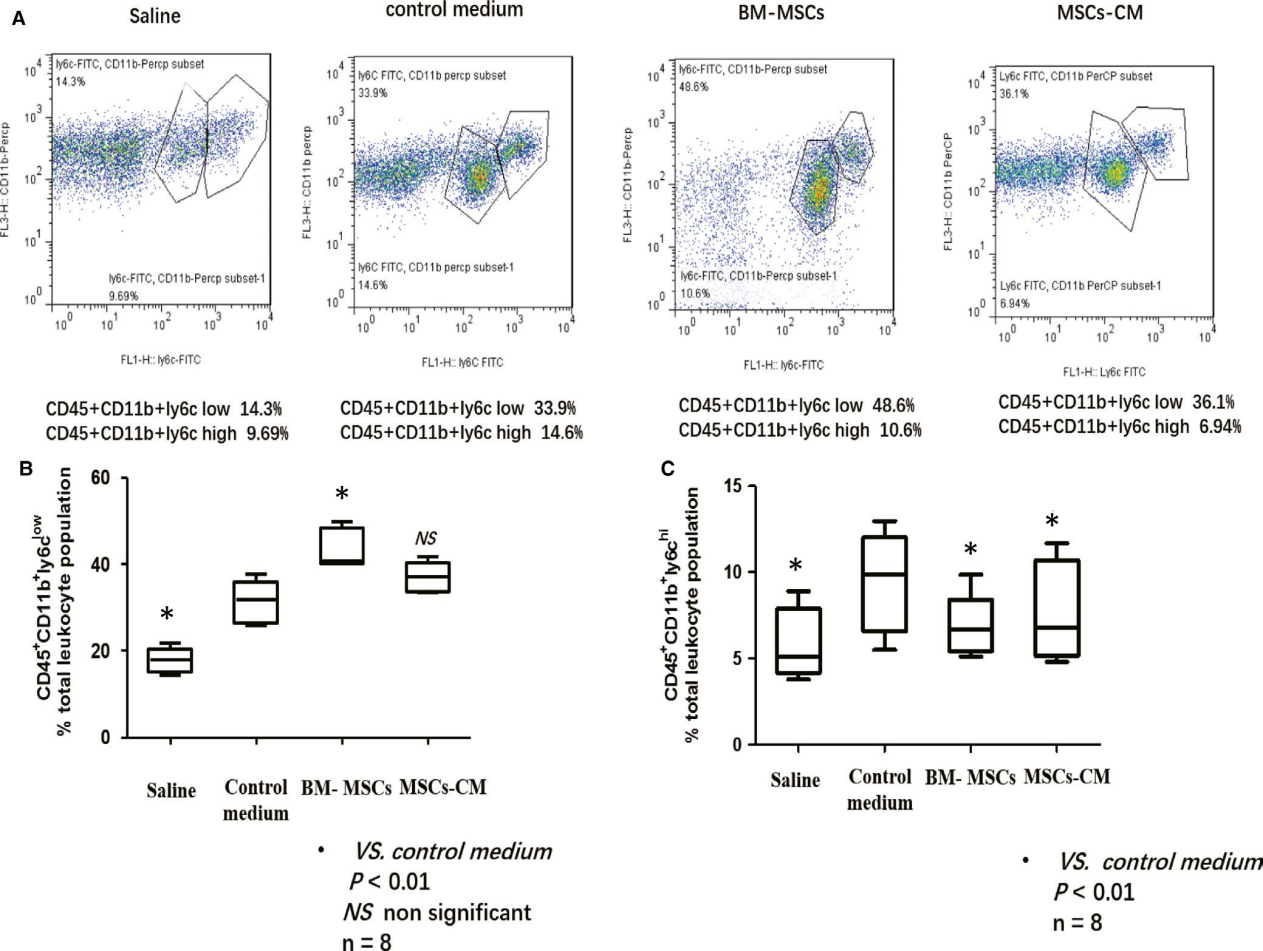
A, Flow cytometry was employed to investigate the subpopulation of circulating monocytes after AngII infusion on day 7 in each group. CD45 + CD11b + Ly6C^high^ population was gated and representative figure was presented. A decrease in CD45 + CD11b + Ly6C^high^ was observed in both BM‐MSC and MSCs‐CM group comparing with control medium group. B, C, Average percentage of CD45 + CD11b + Ly6C^high^ monocytes in total leucocyte population which correlated with inflammation were determined. Significantly reduction in CD45 + CD11b + Ly6C^high^ monocyte proportion was observed in both BM‐MSC and MSCs‐CM group comparing with the control medium group

In the preliminary experiments, we observed that the subpopulation of CD45^+^CD11b^+^Ly6c^high^ monocytes in peripheral blood increased from Day 1 and peaked on Day 7 during AngII infusion (data not shown). In the present study, we measured the CD45^+^CD11b^+^Ly6c^high^ subpopulation of monocytes in peripheral blood on day 7. We found that mean percentage of inflammation‐related monocytes (CD45^+^CD11b^+^Ly6c^high^) among the total leucocytes was significantly decreased in the peripheral blood in the BM‐MSCs (7.567% ± 1.3) and MSCs‐CM (7.323% ± 0.82) group compared with the control medium group (17.98% ± 0.92; Figure 3).

### 3.4 BM‐MSCs and MSCs‐CM increased M2 macrophage and decreased M1 macrophage in aortic aneurysm

**Figure 4:**
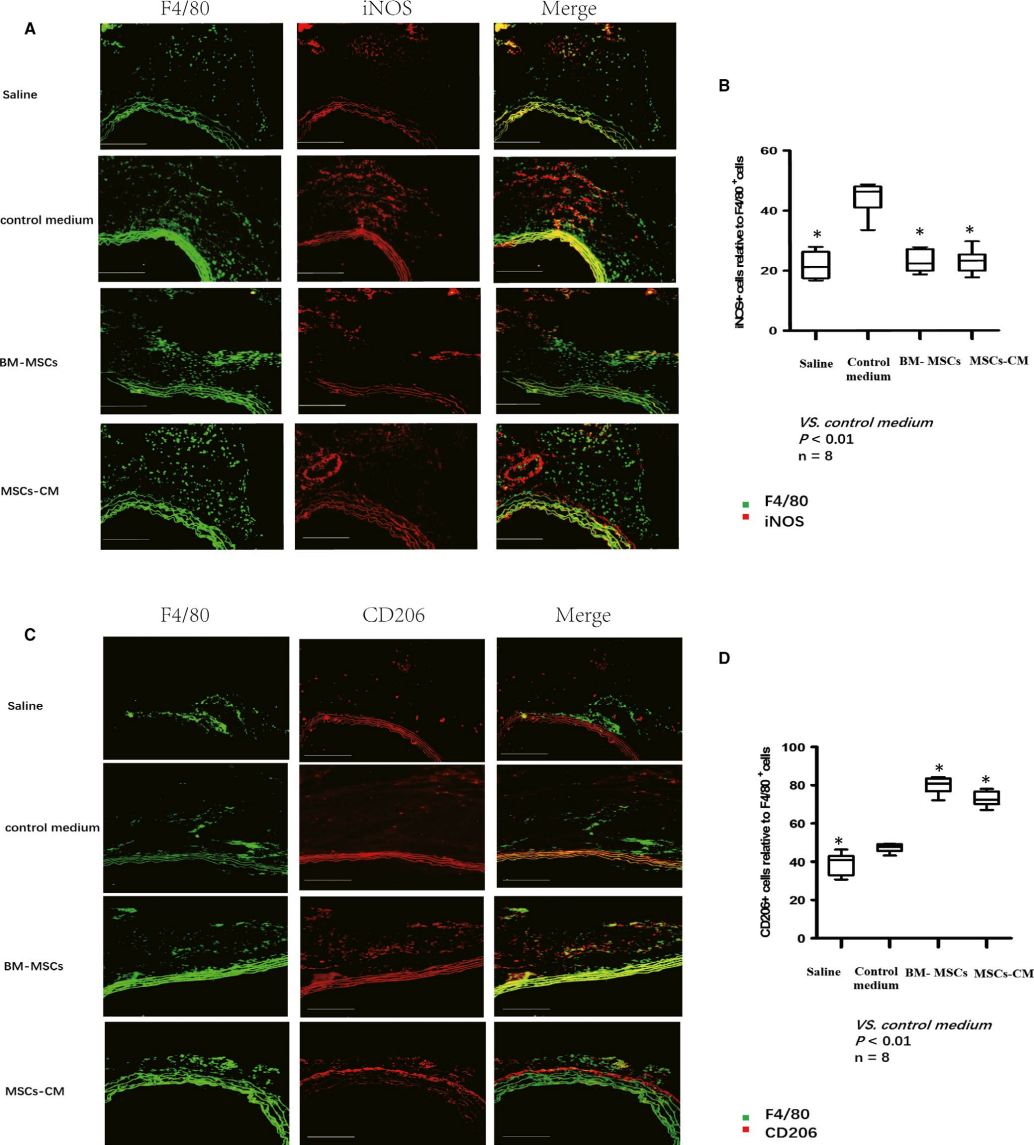
Macrophage polarization at the site of AA. A, M1 macrophage markers (iNOS) and total macrophage F4/80 were observed using immunofluorescence staining in the tissues collected at the site of AA on day 14. A decrease in iNOS+ cells was observed in both BM‐MSC and MSCs‐CM group comparing with the control medium group. B, The average percentage of iNOS+ cells were normalized with total macrophage F4/80+ cells and statistically analysed. Significantly reduction in iNOS+ cells was observed in the BM‐MSC group and MSCs‐CM group comparing with control medium group. C, M2 macrophage markers (CD206) and total macrophage F4/80 were observed using immunofluorescence staining in the tissues collected at the site of AA on day 14. An increase in CD206+ cells was observed in both BM‐MSC and MSCs‐CM group comparing with the control medium group. D, The average percentage of CD206+ cells were normalized with total macrophage F4/80+ cells and statistically analysed. Significantly increase in CD206+ cells was observed in both BM‐MSC and MSCs‐CM group comparing with the control medium group

To further clarify the mechanisms associated with the effects of BM‐MSCs and MSCs‐CM on AngII‐induced AA, AA tissues were collected on day 14. F4/80 (macrophage marker) and iNOS (M1 macrophage marker) were used to detect M1 macrophages in the aortic tissue. The percentage of iNOS+ cells was determined. A decrease in M1 macrophages was observed in the BM‐MSCs (23.07% ± 1.51) and MSCs‐CM group (23.1% ± 1.63) compared with the control medium group (44.37% ± 2.3; Figure 4A,B). F4/80 (macrophage marker) and CD206 (M2 macrophage marker) were used to detect M2 macrophages in the aortic tissue. The percentage of CD206+ cells was determined. An increase in M2 macrophages was observed in the BM‐MSCs (79.93% ± 1.83) and MSCs‐CM (72.9% ± 1.59) group compared with the control medium group (47.3% ± 0.92; Figure 4C,D).

### 3.5 BM‐MSCs and MSCs‐CM inhibited expression of pro‐inflammatory cytokines in AA tissues

**Figure 5:**
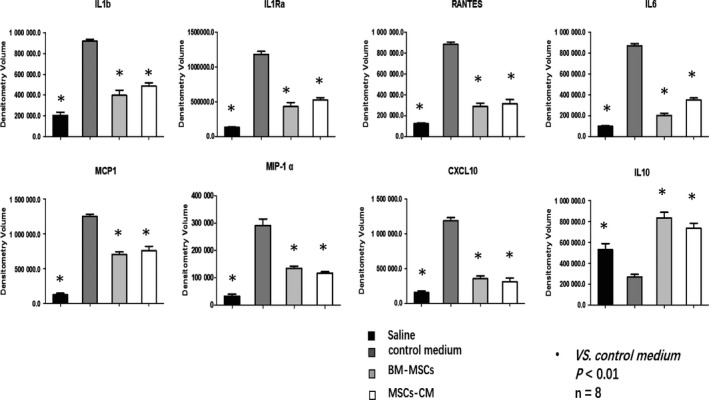
Cytokines in aortic wall. Cytokines related with inflammation were determined in the AA tissues using Proteome Profiler Mouse Cytokine Array Kit. Densitometry volume of each cytokine was determined and statistically analysed. IL‐1b, IL‐1Ra, RANTES, IL‐6, MCP1, MIP‐1α and CXCL10 were all decreased in both BM‐MSC and MSCs‐CM group comparing with the control medium group, while IL‐10 increased in both BM‐MSCs and MSCs‐CM group comparing with the control medium group

Cytokines expression in the AA tissues was determined using the Proteome Profiler Mouse Cytokine Array Kit. Cytokines involved in immune regulatory and chemotactic activities and inflammatory responses (IL‐1b, IL‐1Ra, RANTES (CCL5), IL‐6, MCP1 (CCL2), MIP‐1 (CCL3) and CXCL10) were all decreased in the AA tissues from both BM‐MSCs and MSCs‐CM group compared with the control medium group. The expression of IL‐10 was found to be increased in AA tissues from both BM‐MSCs and MSCs‐CM groups compared with the control medium group (Figure 5).

### 3.6 MSCs‐CM component analysis

Previous studies demonstrated the main components of BM‐MSCs‐CM were IGF1, VEGF, TGF‐β1, FGF basic and SDF1α which play the important biological effects of MSCs.^13^, ^15^ In the present study, the concentration of IGF 1, VEGF, TGF‐β1, FGF basic, SDF1α released in MSCs‐CM was quantified using ELISA (n = 3). IGF‐1, which has growth‐promoting activity, was detected at a higher concentration in MSCs‐CM (1107 ± 316) than in fibroblasts‐CM (212 ± 57; *P* = .0085). VEGF, which induces proliferation and migration of vascular endothelial cells, and is essential for both physiological and pathological angiogenesis, was detected in both MSCs‐CM (479 ± 102) and fibroblasts‐CM (121 ± 51; *P* = .0056). TGF‐β1, which regulates cell proliferation, differentiation and growth, and can modulate expression and activation of other growth factors, was detected in MSCs‐CM (342 ± 27) but undetectable in fibroblasts‐CM. Neither FGF basic nor SDF1α could be detected in MSCs‐CM or in fibroblasts‐CM (Table 2).

**Table 2:** Cytokine secretion in MSCs‐CM and fibroblasts‐CM

Cytokines	MSCs‐CM (pg/mL)	Fibroblasts‐CM (pg/mL)
IGF‐1	1107 ± 316	212 ± 57
VEGF	479 ± 102	121 ± 51
TGF‐β1	342 ± 27	Undetectable
FGF basic	Undetectable	Undetectable
SDF‐1α	Undetectable	Undetectable

### 3.7 BM‐MSCs promoted M2 macrophage polarization in vitro

**Figure 6:**
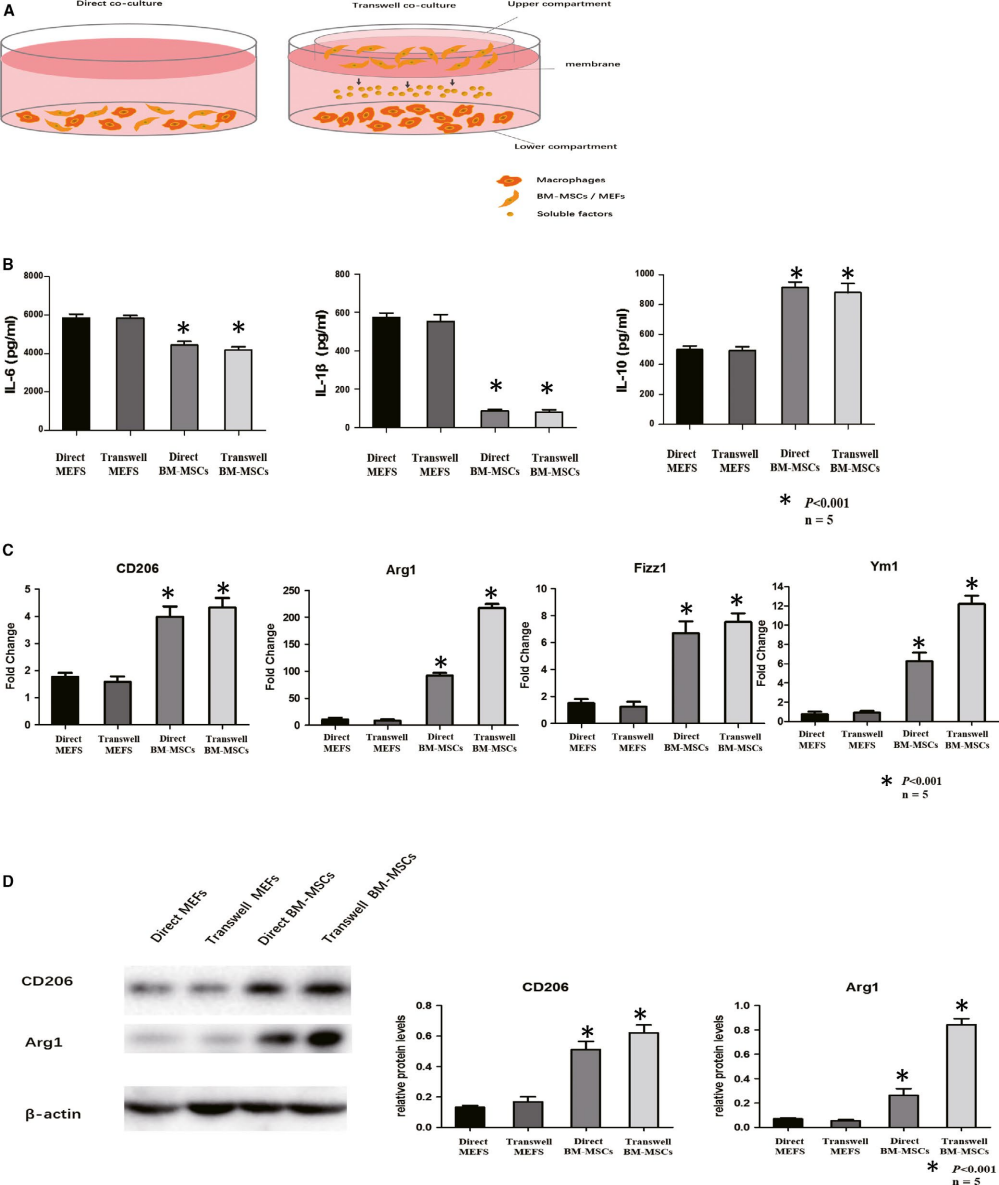
Co‐culturing of macrophages/MEFs and BM‐MSCs. A, Schematic diagram of macrophages directly (left) or transwell (right) co‐cultured with murine embryonic fibroblasts (MEFs)/BM‐MSCs. In the direct co‐culturing system, macrophages were directly contacted with BM‐MSCs/MEFs. In the transwell system, macrophages were cultured in the lower compartment, while BM‐MSCs/MEFs were cultured in the upper compartment. Only soluble factors secreted by BM‐MSCs/MEFs could penetrate the membrane between upper and lower compartment and contacted with macrophages cultured in the lower compartment. Direct contacts between macrophages and BM‐MSCs/MEFs were prevented in the transwell system. B, ELISA was applied to evaluate the content of pro‐inflammation factors (IL‐6, IL‐1β) and anti‐inflammation factors IL‐10 in the supernatant of direct co‐culturing of macrophages with MEFs, transwell co‐culturing macrophages with MEFs, direct co‐culturing macrophages with BM‐MSCs, transwell co‐culturing macrophages with MSCs. IL‐6 were decreased in both direct MSC group and transwell BM‐MSCs group comparing with direct MEFs and transwell MEFs. IL‐1β was significantly decreased in both direct BM‐MSCs group and transwell BM‐MSCs group comparing with direct MEFs and transwell MEFs. On the contrary, IL‐10 was increased in both direct BM‐MSCs and transwell BM‐MSCs group (light grey column) comparing with direct MEFs and transwell MEFs. C, Real‐time PCR was performed to determine the expression of M2 macrophage markers (CD206, Arg1, Fizz1 and Ym1) expressions in direct co‐culturing of macrophages with MEFs, transwell co‐culturing macrophages with MEFs, direct co‐culturing macrophages with BM‐MSCs, transwell co‐culturing macrophages with BM‐MSCs. Expressions of CD206, Arg1, Fizz1 and Ym1 were all increased in both direct BM‐MSCs group and transwell BM‐MSCs group (light grey column) comparing with direct MEFs and transwell MEFs group. D, Western blotting was utilized to analyse CD206 and Arg1 protein expression in direct co‐culturing of macrophages with MEFs), transwell co‐culturing macrophages with MEFs (transwell MEFs), direct co‐culturing macrophages with BM‐MSCs (direct MSCs), transwell co‐culturing macrophages with BM‐MSCs (transwell MSCs). Relative protein levels of CD206 and Arg1 were both determined by normalized with β‐actin expression. CD206 expression and Arg1 expression were increased in both direct BM‐MSCs and transwell BM‐MSCs group comparing with direct MEFs and transwell MEF group

The potential effects of M2 macrophage polarization induced by BM‐MSCs were determined by in vitro co‐culturing of peritoneal macrophages with BM‐MSCs/MEFs (direct contacting with BM‐MSCs/MEF or by transwell device). In the transwell system, only cytokines secreted by BM‐MSCs/MEFs cultured in the upper compartment could pass through the membrane to contact with macrophages cultured in the lower compartment (Figure 6A). Cytokine secretions were determined using ELISA in the supernatant of macrophage cells directed co‐cultured with BM‐MSCs/MEFs (direct co‐culture) or macrophage cells transwell co‐cultured with BM‐MSCs/MEFs (transwell co‐culture). Pro‐inflammatory factors such as IL‐6, IL‐1β were significantly decreased in both direct co‐culture and transwell co‐culture BM‐MSCs group comparing with MEFs group, while anti‐inflammatory factors IL‐10 were increased in both direct co‐culture and transwell co‐culture BM‐MSC group comparing with MEFs group. Significant differences between direct co‐culture and transwell co‐culture supernatants of each group were not observed (Figure 6B). mRNA expressions of M2 macrophage markers were determined macrophage cells directed co‐cultured with BM‐MSCs/MEFs (direct co‐culture) or macrophage cells transwell co‐cultured with BM‐MSCs/MEFs (transwell co‐culture). CD206, Arg1, Fizz1 and Ym1 expressions were all increased in both direct co‐culture and transwell co‐culture supernatants of BM‐MSCs group comparing with MEFs. The abundant of Arg1 expressions was significantly higher in both direct co‐culture and transwell co‐culture supernatants of BM‐MSC group comparing with MEFs (Figure 6C). M2‐related proteins expressions were analysed with Western blotting in each group. CD206 protein expression was up‐regulated in both direct co‐culture and transwell co‐culture cells of BM‐MSC group comparing with the MEF group. As for the Arg1 protein expressions, in the direct co‐culture group, it was slightly increased in the BM‐MSCs group comparing with MEFs group, while in the transwell co‐culture cells, Arg1 expression was significantly up‐regulated in BM‐MSCs group comparing with MEFs group (Figure 6D). In our observation, by co‐culturing with BM‐MSCs, macrophage cells expressed abundant pro‐inflammatory factor IL‐10, and M2 macrophage‐related markers such as CD206, Arg1, Fizz1 and Ym1. The discordant expression of Arg1 in direct co‐cultured macrophage cells with BM‐MSCs and transwell co‐cultured macrophage cell with BM‐MSCs implied that some of the M2 macrophage‐related markers could be effectively induced by BM‐MSCs by direct contacting, some might not.

## 4 DISCUSSION

In the current study, we provide the first evidence that MSCs‐CM suppressed aortic inflammation reaction and prevented AA growth in apoE^−/−^ mice. Administration of MSCs‐CM inhibited MMP‐2 and MMP‐9 expression and protected aortic elastin from damage. These results were associated with a decrease in M1 macrophage and increase M2 macrophage infiltration in AA tissues as well as reduced pro‐inflammatory cytokine expression and induced anti‐inflammatory cytokine expression. We also demonstrated that these effects were comparable with administration of BM‐MSCs. Furthermore, our data in vitro indicated that MSCs‐CM promoted M2 polarization of macrophages. Taken together, our findings indicated that MSCs‐CM can promote macrophage polarize towards M2 phenotype which might suppress inflammatory reactions and enhance subsequent reparative activities, thereby slow AA growth.

BM‐MSCs are a heterogeneous population of fibroblast‐like stromal cells that have been isolated from the bone marrow, fat tissue and umbilical cord. BM‐MSCs have self‐renewing and multipotent properties that can differentiate into adipogenic, chondrogenic and osteogenic lineages. MSC‐based therapeutic approaches seem to be promising in treating a number of diseases. Despite the potential benefits, there still remain several concerns such as the potential risk of immune reactions, insufficient viability of transplanted cells and poor engraftment. Recent studies have shown that BM‐MSCs modulate immune cell function and have cell‐protective effects through the release of specific growth factors, chemokines and cytokines.^13^, ^15^, ^17^, ^21^ Accordingly, direct injection of the supernatant from cultured BM‐MSCs that contains above biological molecules could be an alternative option. Indeed, it has been demonstrated that the administration of MSCs‐CM was as effective as MSC treatment itself in various diseases.^14^, ^15^, ^17^ Recently, MSCs have been shown to prevent AA formation and progression in a variety of AA models.^10^, ^11^, ^12^, ^25^ In the present study, we investigated the effects and mechanisms of the administration of MSCs‐CM on AngII‐induced AA in apoE^−/−^ mice. Our results demonstrated that MSCs‐CM suppressed aortic inflammation reaction and prevented AA growth, which were comparable to BM‐MSCs treatment. Administration of preserved MSCs‐CM is convenient for immediate application and minimizes surgical invasiveness. Furthermore, the use of allogeneic CM can circumvent ethical controversies and immune‐related problems. This therapeutic strategy might be a substantial option for clinical translation in the future.

Macrophages are essential mediators of the inflammatory response and play an important role in inflammation‐associated diseases. Previous studies have suggested that macrophage polarization may be involved in the development of AA.^26^ Recent studies have shown that modulating macrophage polarization could achieve therapeutic effects for AA.^6^, ^7^, ^8^, ^27^ However, it remains unknown whether MSC or MSCs‐CM could suppress vascular inflammation and AA progression through modulating macrophage polarization. In the present study, we observed that the therapeutic effects of BM‐MSCs and MSCs‐CM were associated with decreased M1 macrophages and increased M2 macrophage proportion in AA tissues. Moreover, BM‐MSCs and MSCs‐CM reduced pro‐inflammatory cytokine expression (IL‐1b, IL‐1Ra, IL‐6, etc) and induced anti‐inflammatory cytokine (IL‐10) expression, which inhibits the synthesis of a number of cytokines including IFN‐gamma, IL‐2, IL‐3, TNF‐α and GM‐CSF produced by activated macrophages and by helper T cells. Our results of in vitro study showed that BM‐MSCs and MSCs‐CM have the capacity to modulate M1/M2 macrophage polarization. These data support BM‐MSCs and MSC‐CM could mediate a switch of macrophages into a regulatory anti‐inflammatory M2 phenotype. Our results are in line with several previous studies indicating that MSCs exert anti‐inflammatory properties via macrophage reprogramming.^19^

BM‐MSC secretome analysis studies demonstrated that MSCs‐CM contained high concentrations of IGF, TGF‐β1 and HGF.^15^ Schinkothe et al^13^ screened human MSCs‐CM and categorized these into functional groups: anti‐apoptotic, immunosuppressive, proproliferative and angiogenic modulating. In our study, we found that MSC‐CM contained high levels of IGF1, VEGF and TGF‐β1. In Andre Heinen and colleague's study, they reported that IGF1 treatment improves cardiac remodelling after infarction by inducing M2 polarization.^28^ Wheeler et al^29^ proved that VEGF may contribute to macrophage recruitment and M2 polarization in the decidua. Fang et al^30^ reported that TGF‐β1 could induce M2 polarization in kidney fibrosis. These recent studies connected IGF‐1, VEGF and TGF‐β1 with M2 polarization. It may be one important mechanism to explain that administration of BM‐MSCs or MSCs‐CM could attenuate vascular inflammation and promote an anti‐inflammatory M2 macrophage polarization.

Although our results provided evidence of the therapeutic effects of MSCs‐CM on AA and a novel insight into the mechanisms of MSC‐based AA treatment, several limitations should be mentioned. Although BM‐MSCs and MSCs‐CM mediated M2 macrophage polarization in vitro and we found decreased Ly6c^high^ monocytes in the peripheral blood, decreased M1 macrophages and increased M2 macrophages in AA tissues, however, we did not provide direct evidence on the macrophage phenotypic switch. The treatment with BM‐MSCs and MSCs‐CM may increase recruitment of M2 macrophages in AA tissues, however, it remains unclear whether and how the increased proportion of M2 macrophages actually contributed to aortic repair. Further studies are needed to explore how the BM‐MSC and MSCs‐CM regulate M1/M2 polarization and inhibit AA growth.

## 5 CONCLUSIONS

Taken together, our results demonstrate that MSCs‐CM could effectively alleviate AngII‐induced AA growth in apoE^−/−^ mice, and these protective effects contributed at least partially to modulating M1/M2 macrophage polarization. These findings may provide a novel insight into the mechanisms of BM‐MSCs based treatment for AA. MSCs‐CM could be a more optimized option for treating AA and resolving the clinical application disadvantages associated with BM‐MSCs transplantation. We believe that MSCs‐CM may serve as a basis of a novel cell‐free therapeutic approach for AA.

## CONFLICT OF INTEREST

The authors declare that they have no competing interests.

## AUTHOR CONTRIBUTIONS

XMZ and XMF designed the experiment and interpreted results. YZZ and ZC drafted manuscript. YZZ, CZ, YW, QYW and YZ performed experiments. XBL, JML, XMF and XMZ made critical revision to manuscript. All authors have read and approved the final manuscript. XMZ, and XMF are co‐corresponding authors.

## Data Availability

All data generated or analysed during this study are included in this article.
